# An efficient bacterial laccase-mediated system for polyurethane foam degradation

**DOI:** 10.3389/fmicb.2025.1638208

**Published:** 2025-08-25

**Authors:** Xiaomin Zhu, Youren Duan, Jianqi Lu, Wei Xia, Yujia Peng, Jiawei Liu, Weiliang Dong, Min Jiang

**Affiliations:** ^1^Key Laboratory for Waste Plastics Biocatalytic Degradation and Recycling, College of Biotechnology and Pharmaceutical Engineering, Nanjing Tech University, Nanjing, China; ^2^State Key Laboratory of Materials-Oriented Chemical Engineering, Nanjing Tech University, Nanjing, China

**Keywords:** polyurethane, bacterial laccase CotA, ABTS, degradation mechanism, color shift

## Abstract

Polyurethane (PU), a segmented block copolymer with chemically resistant urethane linkages and tunable architecture, presents persistent biological recycling challenges. This study presents a Bacterial Laccase-Mediated System (BLMS) derived from *Bacillus subtilis* for efficient degradation of polyester- and polyether-PU. Utilizing the laccase CotA and mediator 2,2'-azino-bis (3-ethylbenzothiazoline-6-sulfonic acid) (ABTS), the BLMS demonstrated effective de polymerization of both commercial and self-synthesized PU foams, including polyester- and polyether-types. The weight loss of the self-synthesized polyester-foam and the commercial polyether-foam reached up to 21.24 ± 1.20% and 3.81 ± 0.36%, respectively. Subsequently, we detected oxygenated products such as ketones, alcohols, aldehydes, acids, esters, ethers, and 2,4– toluenediamine (2,4-TDA) indicating that bacterial laccase CotA exhibited redox catalytic activity toward PU. Moreover, an interesting phenomenon was observed during the degradation process that the solution turned purple. We predicted that this attribute to the enzymatic oxidation of ABTS to the radical cation ABTS^·+^, which subsequently reacts with 2,4-TDA to form the purple product. This study finds a plastic degrading enzyme capable of hydrolyzing urethane bonds in PU, offering a promising contribution to the development of a bio-based circular economy for PU biodegradation and recycling.

## Graphical Abstract

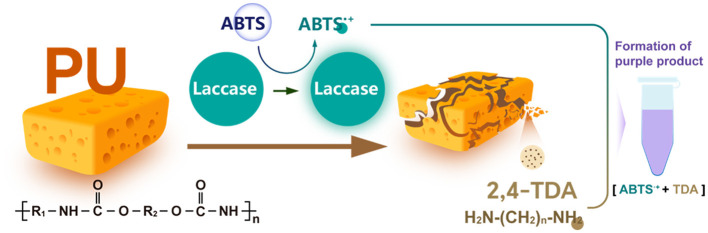

## 1 Introduction

Polyurethane (PU), a class of polymer materials characterized by repeating urethane bond in their main chain, has been widely utilized in various industries since its industrial-scale production began in the mid-twentieth century (Millet et al., 2018). Owing to its exceptional mechanical properties-such as wear resistance, oil resistance, and aging resistance-PU has found extensive applications in fields including building insulation, automotive interiors, furniture manufacturing, footwear production, and packaging materials (Pierre and Luc, 2018). In 2022, PU accounted for 5.3% of global plastic production, with an estimated usage of around 20 million tons (Report, 2023). PU can be categorized into thermoplastic PU and thermosetting PU, depending on the degree of molecular crosslinking (Skleničková et al., 2020). Thermoplastic PU, characterized by a linear or slightly crosslinked branched structure, exhibits excellent process ability and flexibility. In contrast, thermosetting PU is highly crosslinked, insoluble in water, and does not melt at high temperatures, making it resistant to reshaping through processing (Skleničková et al., 2020). PU foam, including both flexible and rigid foam, accounts for 60%−70% of the global PU market in 2021, representing a significant proportion of PU products worldwide (Research, 2021).

Due to their chemical inertness and highly cross-linked polymer structure of PU materials, exhibit extremely low biodegradability in natural environments. This results in the long-term accumulation of waste in soil and aquatic ecosystems, contributing to the formation of white pollution (Temporiti et al., 2022). To address this issue, researchers have explored various disposal technologies for waste PU, including physical, chemical, and biological recycling methods. However, physical and chemical techniques suffer from several drawbacks, such as high energy consumption, secondary pollution, and limited applicability to complex PU waste streams (Istrate et al., 2021). In contrast, biological recycling, as an emerging green approach, utilizes enzymatic catalysis to depolymerize PU. This method offers significant advantages, including mild reaction conditions (ambient temperature and pressure), high substrate selectivity, and tunable degradation products, making it a promising solution for sustainable PU waste management (Liu et al., 2021; Magnin et al., 2021). The biodegradation of PU can be achieved through the use of insects (Guo et al., 2019; Liu et al., 2022), microorganisms (Liu et al., 2024b; Magnin et al., 2020), or enzymes (Liu et al., 2021, 2024a). Among these methods, the use of enzymes to depolymerize PU into monomers for re polymerization is the most effective approach for recycling (Liu et al., 2021). Hydrolases and oxidoreductases are the two types of enzymes that have been reported to degrade PU. Hydrolases, including cutinases (LCC, HiC, TfCut2, Tcur1278, Tcur0390) (Schmidt et al., 2017), esterases (PudA, PulA, E3567) (Nakajima-Kambe et al., 1997; Vega et al., 1999), and lipases (PueA, PueB) (Magnin et al., 2019), are the most active during PU de polymerization. However, their de polymerization principle mainly involves the ester bond acting on the polyol segment. This means that they can only depolymerize polyester-based PU and are not effective against polyether-based PU. A recent study showed that a Fungal Laccase-Mediated System (FLMS), which belongs to oxidoreductases, was effective in degrading polyester-based and polyether-based PU foams which were self-synthesized (Magnin et al., 2021). Currently, there are no relevant reports available on the effectiveness of Bacterial Laccase-Mediated System (BLMS) on PU degradation, particularly on polyether-based PU foams. Although, BLMS has demonstrated excellent degradability for Poly Styrene (PS) and Poly Ethylene (PE) plastics due to its stronger environmental tolerance (He et al., 2023). Further research is needed to investigate the effectiveness of BLMS on PU degradation and to explore its potential as a solution for PU waste management.

In our previous research, a BLMS derived from *Bacillus subtilis* was constructed and applied in hydroquinone determination and dye de colorization. The results showed that the BLMS exhibited more stable enzymatic ability compared to FLMS (Zhang et al., 2018). The objective of this study is to examine the possible utilization of BLMS in the degradation of thermoset PU, as well as to investigate the occurrence of purple substances during the degradation process. Degradation assays were conducted using commercial and self-synthesized PU foam as the substrate. The degradation process was performed using BLMS with [2,2'-azino-bis (3-ethylbenzothiazoline-6-sulphonic acid)] ABTS as mediator. Besides, the modification in materistics in diverse PUs after degradation was performed using Scanning Electron Microscope (SEM), Fourier Transform Infrared Spectroscopy (FTIR) and Thermogravimetric Analysis (TGA). Furthermore, the degradation products were identified by gas Chromatography-Mass Spectrometry (GC-MS), and the potential mechanism underlying the purple coloration of the solution during the degradation process was investigated.

## 2 Material and methods

### 2.1 Strains, plasmids, and chemicals

The recombinant strain *E. coli* BL21 (DE3)-pET28a (+)-*cotA* was previously constructed in the laboratory. The commercial foams (polyester-CPU and polyether-CPU) derived from sofa cushions were purchased in Nantong Dagong Co., Ltd. The polyethylene glycol (PEG, M_w_ ≈ 2000) and Poly Capro Lactone (PCL, M_w_ ≈ 2000) was purchased from Aladdin Reagent Co. Ltd. (Shanghai, China). 2,4-TDI, ABTS were purchased from Sinopharm Chemical Reagent Co, Ltd. (Shanghai, China). All chemicals were of analytical grade. Other molecular reagents were purchased from Takara Bio Inc. (Shiga, Japan).

### 2.2 Protein expression and purification

The *E. coli* BL21 (DE3)-pET-28a (+)-*cotA* were grown in LB medium at 37°C to an OD_600_ of 0.6, then induced by 0.1 mm isopropyl β-D-thiogalactopyranoside (IPTG) at 16°C for 24 h. Subsequently, the cells were harvested at 6,000 rpm and lysed by ultrasonic disruptor. After centrifugation at 8,000 rpm for 15 m, the supernatant from the lysate was collected for subsequent purification with a pre-equilibrated nickel Nitrilotriacetic Acid (Ni-NTA) column (Cytiva, Sweden). The column was washed using lysis buffer supplemented with 30 mm imidazole and eluted using lysis buffer supplemented with 300 mm imidazole. The purity of the eluted protein is assessed by SDS-PAGE analysis. The laccase assay was performed using ABTS (final concentration, 5 mm) as a substrate in 0.1 m sodium acetate buffer, at pH 4.0. The OD of the oxidation product was determined at 420 nm (ε_420_ = 36,000/m/cm). One unit of enzymatic activity was defined as the amount of enzyme that oxidized 1 mm of ABTS per m.

### 2.3 Synthesis of polyester-type and polyether-PU foams

One hundred gram of PEG or PCL were dissolved in tetrahydrofuran (THF) solvent. After that, 25 g of 2,4-TDI were poured into the mixture and stir vigorously to ensure thorough mixing. Add a few drops of catalyst to the reaction solution and continue stirring for 20 m. The prepared solution was transferred into a reactor and rapidly heat it to 120°C. During this process, solvent evaporated, and the temperature was maintained for 30 m to obtain a white foamy solid, yielding thermosetting polyether- or polyester-PU foam, respectively.

### 2.4 Degradation assay of different types of PU by laccase CotA

The commercial foam (CPU) was cut into 1 × 1 × 1 cm pieces (with the weight controlled within 50 mg ± 5 mg), while the self-synthesized foam (SPU), though irregular in shape, was also controlled to a weight of 50 mg ± 5 mg for subsequent degradation experiments. The degradation experiments were conducted in a total reaction volume of 5 mL. The degradation system contained 0.125 mg of laccase CotA and ABTS at a final concentration of 0.02 mM, with the volume adjusted to 5 mL using sodium acetate buffer (0.1 M, pH 4.0). The experiments were-incubated at 30°C with shaking at 200 rpm for 10 d and performed in triplicate. Fresh enzyme solution was replenished on days 3, 6, and 9 to maintain enzyme activity. The degradation process was monitored and recorded on days 4, 7, and 10. On the 10th d, all the foams were recycled.

### 2.5 Characterization of PU material properties before and after degradation

The degraded foams were sequentially washed with 1% SDS, ethanol, and deionized water to remove enzyme. Subsequently, foams were dried at 60°C until a constant weight was achieved for the measure of weight loss. All samples were rinsed with 1% SDS, then rinsed with Milli-Q water and ethanol, sputter-coated with Au. SEM was conducted using a Hitachi SU8010 field-emission microscope (Hitachi High-Technologies, Japan) operated in low-vacuum mode (30–50 Pa). Secondary electron images were acquired at 5 kV accelerating voltage with probe currents of 10-100 pA. FTIR spectra were recorded on a Nicolet iN10 spectrometer (Thermo Fisher Scientific, USA) equipped with a diamond universal attenuated total reflection (UATR) module and liquid nitrogen-cooled MCT/A detector. Spectra were collected in the 4,000–500 cm^−1^ range with 4 cm^−1^ resolution (32 scans/spectrum), preceded by atmospheric background subtraction under identical acquisition conditions. TGA was performed using a NETZSCH TG 209 F3 Tarsus system (NETZSCH-Gerätebau GmbH, Germany) with precisely weighed samples (~4.0 ± 0.2 mg) heated from 50°C to 700°C at 20°C/min under 50 mL/min N_2_ purge (99.999% purity). Instrument control and data processing were executed through manufacturer-supplied software packages (SEM: PC-SEM v6.02; FTIR: OMNIC v9.2.38; TGA: Proteus v6.1.0).

### 2.6 Identification of products during the degradation process of PU

The supernatant obtained after centrifugation of the 10-d degradation reaction was analyzed by HPLC to determine the degradation products. The degraded foam was sequentially washed with 1% SDS, ethanol, and deionized water to remove residual substances such as enzymes. Subsequently, it was dried at 60°C until a constant weight was achieved to determine the final mass. The initial mass of the PU foam was recorded as m_1_. HPLC analysis was performed on an HPLC 1260 system (Agilent) equipped with a C 18 column (10 × 4.6 mm, Agilent Technologies). The mobile phase was composed of 10% buffer A (acetonitrile) and 90% buffer B (H_2_O). The flow rate was 1 mL/min. Separation was performed at 30°C with detection at 240 nm.

GC-MS analysis was performed using a TRACE1300-ISQ7000 GC-MS system (Thermo Fisher Scientific, USA). Chromatographic separation was achieved on a DB-5MS capillary column (30.0 m × 0.25 mm, 0.25 μm film thickness) with the following temperature program: initial temperature 50°C (held for 4 m), ramped at 10°C/min to 270°C, and held for 9 m. The injector temperature was maintained at 300°C with a split ratio of 10:1, using helium as carrier gas at a constant flow rate of 1.0 mL/min. Sample injection volume was 1 μL, and the transfer line temperature was set to 280°C. Mass spectrometric detection employed electron ionization (EI) at 70 eV with an ion source temperature of 280°C. Data acquisition utilized full scan mode over a mass range of 35–600 m/z, with a solvent delay of 3.8 m. The identification of unknown compounds derived from the reaction was achieved by comparison of mass spectra with database.

### 2.7 Exploration of the purple substance formation during degradation process

A 10 mm stock solution of 2,4-TDA was prepared using methanol, and a 0.04 mm stock solution of ABTS was prepared using deionized water. To generate the radical cation ABTS^·+^, 0.08 mm ABTS was mixed with 1 M Ammonium Persulfate (AP) in a 1:1 (v/v) ratio and allowed to react at room temperature in the dark for 30 m to ensure complete oxidation. Subsequently, three experimental groups were set up as follows: (1) 500 μL of the ABTS stock solution was mixed with 500 μL of 0.5 mm 2,4-TDA in a 1:1 ratio, resulting in a total reaction volume of 1 mL; (2) 500 μL of the ABTS stock solution, 25 μg of laccase, and 500 μL of 0.5 mm 2,4-TDA were mixed in a 1:1 ratio, yielding a total reaction volume of 1 mL; (3) 500 μL of the oxidized ABTS^·+^ solution was mixed with 500 μL of 0.5 mm 2,4-TDA in a 1:1 ratio, resulting in a total reaction volume of 1 ml. The color changes in the three experimental groups were observed and recorded. Next, the aforementioned samples were used as the experimental groups, and solution of 2,4-TDA, ABTS, and ABTS^·+^ served as controls, respectively. UV scanning was performed to observe changes in the peak patterns among the samples. Subsequently, a gradient of 2,4-TDA solutions (0.05–0.4 mm, in increments of 0.05 mm) was prepared and mixed with oxidized ABTS^·+^ (0.04 mm) in a 1:1 ratio. A standard curve correlating absorbance with 2,4-TDA concentration was established based on the relationship between 2,4-TDA concentration and wavelength.

## 3 Results

### 3.1 Expression and purification of laccase CotA

Following induction of expression, recombinant CotA from *E. coli* BL21 (DE3) were purified. This resulted in an intracellular enzyme protein expression level of 0.25 mg/mL with a specific activity of 8.38 U/mg. SDS-PAGE analysis showed the successful soluble expression of laccase in *E. coli* BL21 (DE3), with the molecular weight of the expressed protein band ranging between 55 kDa and 70 kDa, consistent with the theoretical size of laccase (65.0 kDa). A single protein band was observed and washed from the elution buffers containing 200 mm, 250 mm, and 300 mm imidazole at pH 8.0, respectively (Supplementary Figure S1). The purified enzyme eluted under these conditions were subsequently desalted and concentrated for use in BLMS degradation experiments.

### 3.2 Degradation assay of different types of PU by bacterial laccase-mediated system

**Figure 1:**
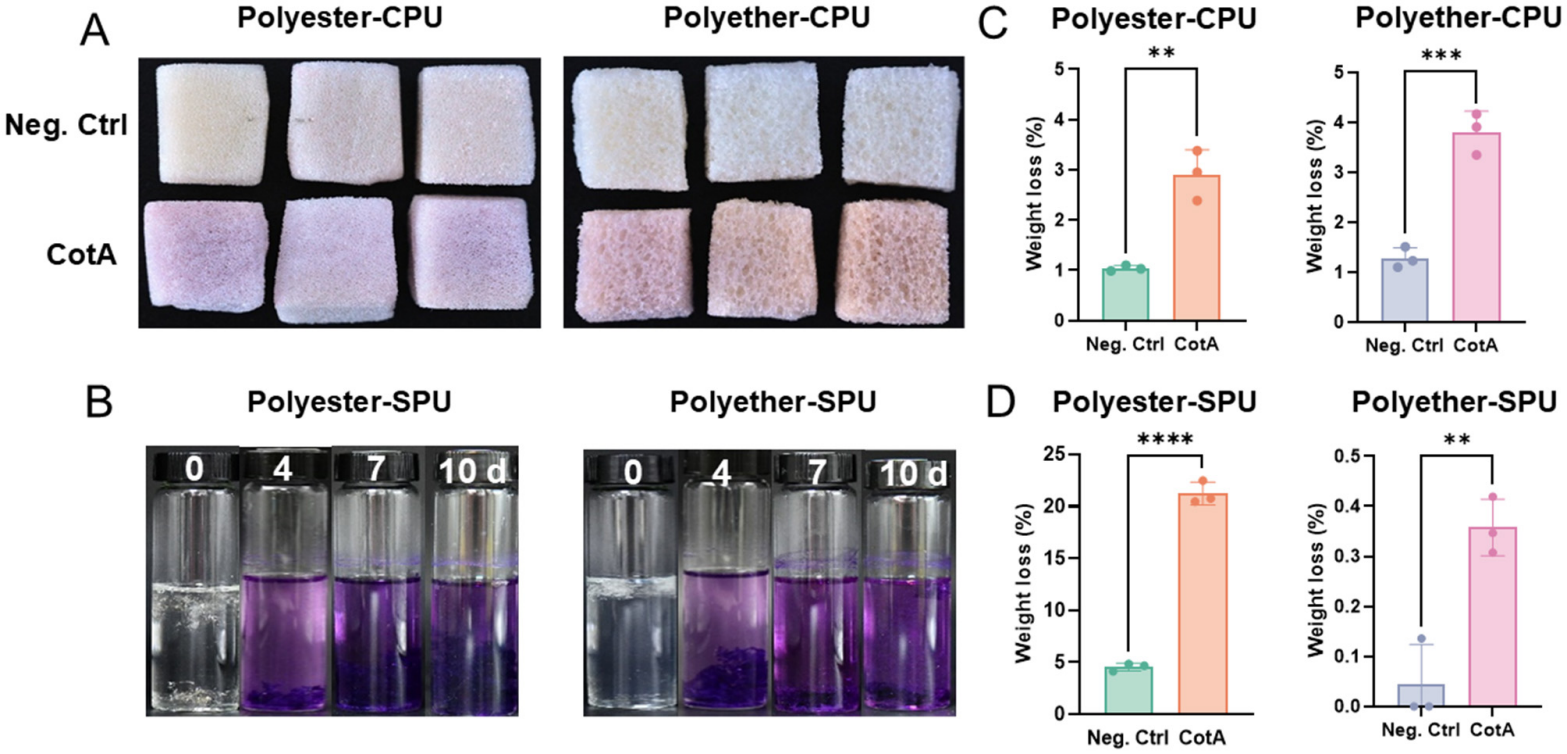
Enzymatic hydrolysis evaluation of PU foams by BLMS. **(A)** Physical diagram of commercial PU foams (CPU): polyester-CPU (left) vs. polyether-CPU (right) after 10 d treatment with BLMS. **(B)** Physical diagram of self-synthesized PU foams (SPU): polyester-SPU (left) vs. polyether-SPU (right) after 10 d treatment with BLMS. **(C)** Weight loss of CPU (*n* = 3). **(D)** Weight loss of SPU (*n* = 3). Statistical significance was assessed by unpaired two-tailed *t*-test. *P* < 0.01 for all comparisons; ^**^, ^***^, ^****^indicate levels of significance.

We initially employed the BLMS system to degrade Commercial PU Foam (CPU). It was observed that the solution of degradation system turned purple, and the surface of foam also exhibited a purple coloration, regardless of whether polyester-based CPU or polyether-based CPU was used (Figure 1A). As Subsequently, self-synthesized PU foams (SPU) were also used for degradation assay and found that the solution also turned purple during its degradation process by the BLMS system (Figure 1B). Furthermore, throughout the 10-d degradation process, the degradation solution exhibited a progressively intensifying purple coloration as the degradation duration increased. These results revealed a common phenomenon of purple coloration associated with the degradation of PU foam by the BLMS system.

When commercial PU foam was used as the substrate, the BLMS system achieved weight loss of 2.90% ± 0.50% and 3.81% ± 0.36% for polyester- and polyether-CPU, respectively (Figure 1C). When the self-synthesized foam served as the substrate, the weight loss for polyester- and polyether- SPU reached 21.24 ± 1.20% and 0.36% ± 0.06%, respectively (Figure 1D). Statistical significance was evaluated using unpaired t-tests. The resulting *p-values* for polyester-CPU and polyether-CPU (Figure 1C), as well as polyester-SPU and polyether-SPU (Figure 1D), were 0.0029, 0.0007, 0.0001, and 0.005, respectively. According to established statistical criteria (*p* < 0.01), these differences were considered statistically significant. It was observed that the BLMS system exhibited a higher degradation capability for the self-synthesized polyester- SPU, whereas the weight loss for the polyether-SPU and the commercial foam was not significant. However, it is an interesting phenomenon that the solution turns purple during the degradation process using different PUs, as it suggested the formation of products released during degradation process. Therefore, it is certain that all PU substrates have undergone varying degrees of degradation, although the weight loss was not detectable. Consequently, further degradation characterization needs to be carried out using other material characterization methods to detect traces of degradation.

### 3.3 Modifications in material characteristics in diverse PUs after degradation

**Figure 2:**
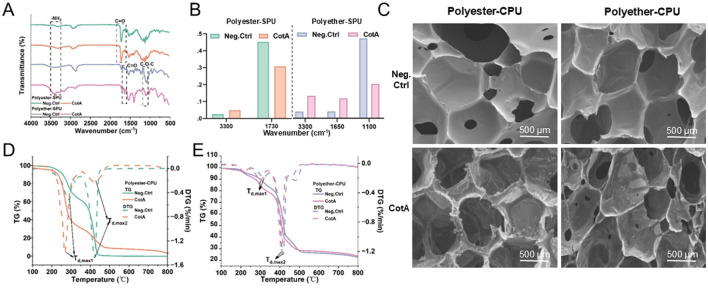
Modifications in material characteristics in PU foams treated by BLMS. **(A, B)** FTIR spectra of SPU. **(C)** SEM of CPU. **(D, E)** TG/DTG curves of CPU.

Subsequently, the modification of material properties using SEM, FTIR, and TGA were analyzed. FTIR was used to analyze modified functional groups in the enzyme-treated polymer. The FTIR spectrum of the BLMS-treated both SPU revealed a significant increase in the peak at 3,300 cm^−1^ for polyether-CPU, and a slight increase at 3,300 cm^−1^ for polyester-CPU, which corresponds to the stretching of N-H and respresent free amine group (-NH_2_) (Figures 2A, B) (Magnin et al., 2021). The result demonstrate that hydrolysis of urethane bonds occurred in PU, and the extent of degradation of this bond was higher in polyether-SPU compared to polyester-SPU. For the BLMS-treated polyester-SPU, the FTIR spectrum showed decrease in the peak at 1,730 cm^−1^, which corresponds to the carbonyl groups (C=O), indicating the hydrolysis of ester bonds (Di Bisceglie et al., 2022). The peak of BLMS-treated polyether-CPU at 1,100 cm^−1^ which corresponds to the C-O-C elongation were significantly reduced, indicating that the ether bond was broken through oxidation by CotA (Magnin et al., 2021). For the BLMS-treated polyether-SPU, the FTIR spectrum showed an increase in the peak at 1,650 cm^−1^, which corresponds to the carbonyl groups (C=O). The intensified C=O stretching vibration at 1,650 cm^−1^ and the attenuated C-O-C stretching mode at 1,100 cm^−1^ collectively indicate that oxidative degradation of polyether chains induces ether bond cleavage, with concomitant generation of carbonyl compounds such as ketones or aldehydes (Magnin et al., 2021; Yao et al., 2022).

SEM can be used to showed the morphological changes of the BLMS treated foam (Figure 2C). It can be observed that the negative control of two commercial PU foams (without BLMS treatment) exhibits intact and smooth structures and surfaces. In contrast, the skeletons of both types of foams treated with BLMS are severely damaged, with their internal structures becoming rough and uneven. Notably, the polyester-CPU foam exhibits more significant erosion marks on its skeleton. Compared to the polyester-CPU, the polyether-CPU foam displays more significant and extensive holes within its internal structure. These results indicate that both types of PU foams are significantly attacked by the enzymes.

TGA is a thermal analysis technique that measures sample mass changes over time or temperature, used to assess thermal stability and material composition (Ha and Jeon, 2024). It is employed to study the thermal stability and composition of materials. Thermal parameters, such as the temperature at 5% weight loss (T_5%_), 50% weight loss (T_50%_) and maximum decomposition temperature (T_d, max1_ and T_d, max2_) generally used to evaluate the thermal properties of the materials (Kumar et al., 2022). In contrast to the untreated control polyester-CPU sample (T_5%_ 229.1°C, T_50%_ = 393.1°C, T_max1_ = 295.1°C, T_max2_ = 420.6°C), all investigated parameters were found to be lower in the BLMS-treated (T_5%_ = 222.3 °C, T_50%_ = 305.3°C, T_max1_ = 289.0 °C, T_max2_ = 419.8°C) (Figure 2D). In contrast to the untreated control polyether-CPU sample (T_5%_ = 233.2°C, T_50%_ = 419.2°C, T_max1_ = 330.1°C, T_max2_ = 412.7°C), all investigated parameters were found to be lower in the BLMS-treated (T_5%_ = 212.3°C, T_50%_ = 407.3°C, T_max1_ = 321.6°C, T_max2_ = 410.4°C) (Figure 2E). This indicates a decreased thermal stability of the polymer as a result of enzymatic degradation.

### 3.4 Identification of degradation products and mechanisms underlying purple coloration in PU degradation

These detected degradation products were mainly oxygenated products such as ketones, alcohols, aldehydes, acids, esters, and ethers in terms of product type by GC-MS analysis, and the carbon chain lengths were mainly concentrated between C2 and C16 (Table 1). GC-MS analysis revealed that the degradation products of the two substrates partially overlapped. When polyester-SPU was used as the substrate, oxidative cleavage was observed not only in the hard segments (isocyanate blocks) of the PU but also in the soft segments (polyol blocks), where multiple oxidative degradation products were detected. In contrast, when polyether-SPU served as the substrate, the variety of degradation products in the soft segments was significantly lower. These results clearly indicate that laccase exhibits stronger redox catalytic activity toward polyester-SPU than polyether-SPU, suggesting that ester bonds are more susceptible to redox reactions and subsequent degradation than ether bonds.

**Table 1:** Products of the enzymatic treatment of PU using the laccase-mediator system of CotA, as detected by GC-MS analysis.

**Polyester–SPU**			**Polyether–SPU**		
**Name**	**Formula**	**Compound structure**	**Name**	**Formula**	**Compound structure**
Acetic acid, methyl ester	C3H6O2	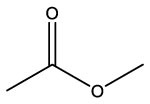	Acetic acid, methyl ester	C3H6O2	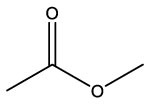
2-Propanone, 1–hydroxyl	C3H6O2	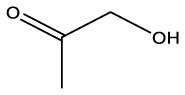	2–Propanone, 1–hydroxyl	C3H6O2	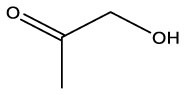
Acetic acid, hydrazide	C2H6N2O	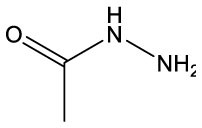	Acetic acid, hydrazide	C2H6N2O	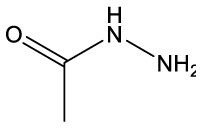
Acetic acid	C2H4O2	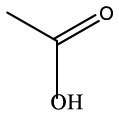	Acetic acid	C2H4O2	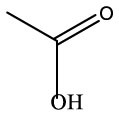
Nitrous acid, ethyl ester	C2H5NO2	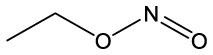	Nitrous acid, ethyl ester	C2H5NO2	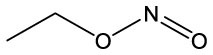
Benzene, 1–methoxy−2–(methoxymethyl)	C9H12O2	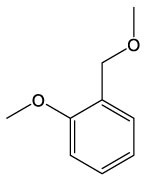	Butanamide, 2–hydroxy–N,3,3–trimethyl	C7H15NO2	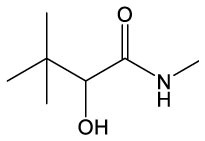
Benzeneethanol, 2–methoxy	C9H12O2	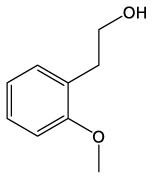	4–O–Methyl–d–arabinose	C6H12O5	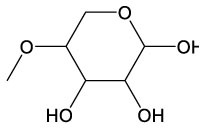
3,4 Dehydro–dl–proline	C5H7NO2	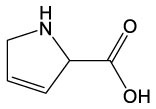	6–Hydroxyeugenol	C10H12O3	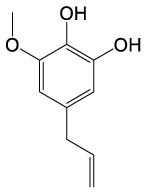
3,6,9,12–Tetraoxatetradecan−1–ol	C10H22O5	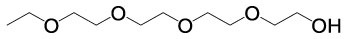	2,3,4–Trimethoxycinnamic acid	C12H14O5	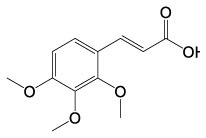
Ethanol, 2–[2–(2–ethoxyethoxy) ethoxy]	C8H18O4	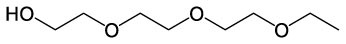			
2,5,8,11,14–Pentaoxahexadecan−16–ol	C11H24O6	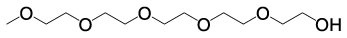			
Tetraethylene glycol diethyl ether	C12H26O5	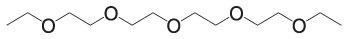			
Heptaethylene glycol	C14H30O8	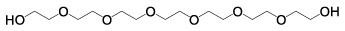			
2–[2–[2–[2–[2–[2–[2–(2–Hydroxyethoxy) ethoxy]ethoxy]ethoxy]ethoxy] ethoxy]ethoxy]ethanol	C16H34O9	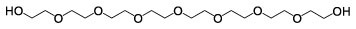			

**Figure 3:**
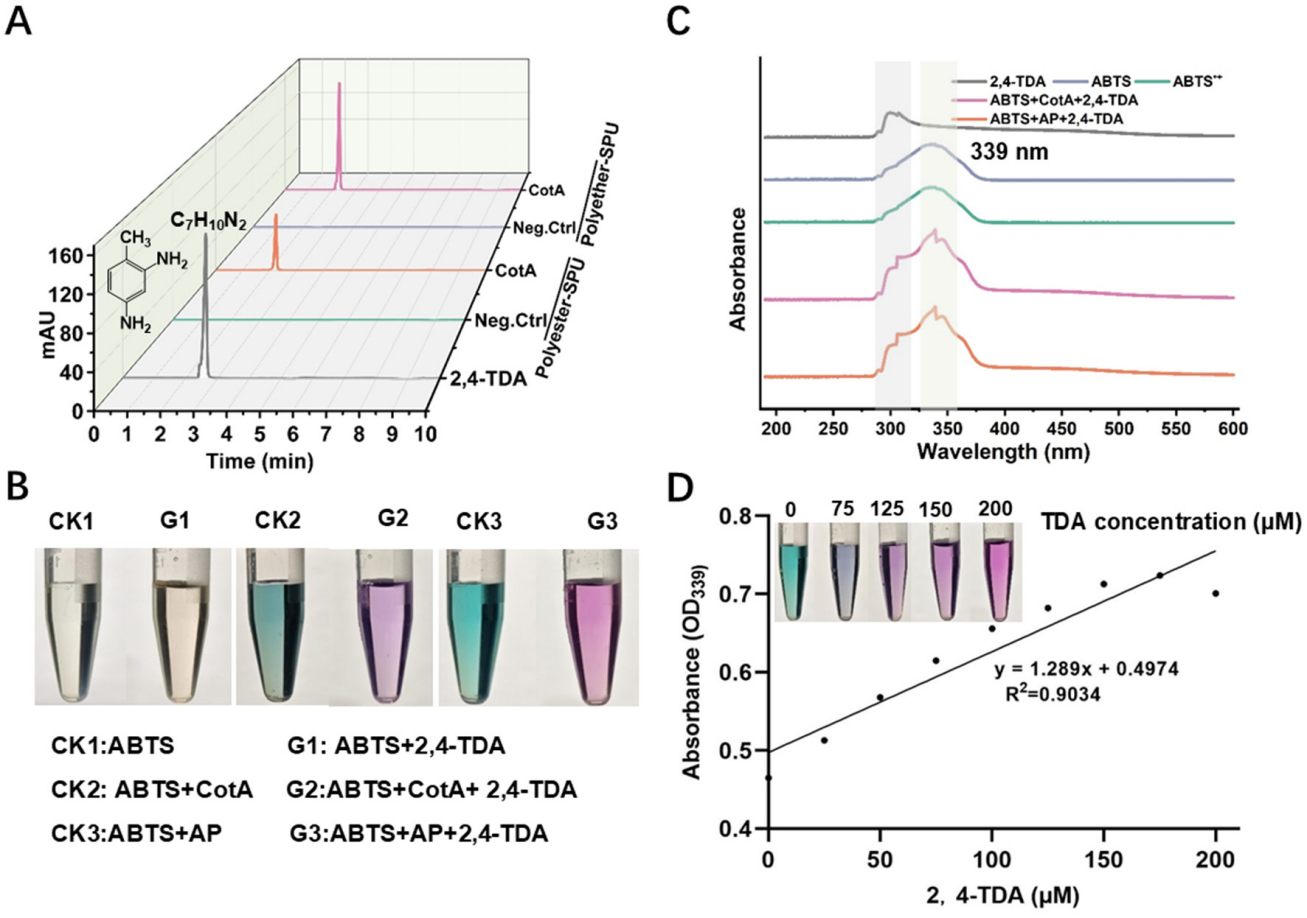
Identification of degradation products and mechanistic insights into purple discoloration during PU degradation. **(A)** HPLC analysis of the degradation product. **(B)** Investigation of the causes of purple discoloration in solution. CK1: ABTS; G1: ABTS+2,4-TDA. CK2: ABTS+ CotA; G2: ABTS+CotA+2,4-TDA. CK3: ABTS+ Ammonium persulfateAP; G3: ABTS+AP+2,4-TDA. **(C)** Full-wavelength UV-vis spectra (190-600 nm) of the above samples. **(D)** Correlation analysis of purple discoloration (OD = 339) in ABTS^·+^ reacted with varying concentrations of 2,4-TDA (*R*^2^ = 0.9034).

As the SPUs were self-synthesized, its structure was clear. Therefore, we used the SPUs as substrates for identification of products during the degradation process. FTIR analysis reveals the cleavage of urethane bonds in PU, suggesting the generation of diamine compounds. Based on the use of 2,4-TDI as the synthetic precursor, 2,4-TDA is thought as a definitive degradation product. Further, we detected the product 2,4-TDI during the degradation of both SPU using HPLC (Figure 3A). It can be observed that 2,4-TDI was not detected in the control groups (without enzyme) of both SPUs. After adding the enzyme, 2,4-TDA was detected in the degradation solutions of both substrates. When using polyester- and polyether-SPU as the substrate, 0.5 mm and 1 mm concentration of 2,4-TDA was produced during the degradation process for each material, respectively. We observed that the concentration of 2,4-TDA released from polyether-SPU was 2 times higher than from polyester-SPU. This result is consistent with FTIR spectrum.

The product 2,4-TDA were speculated that related to the mechanism behind the formation of the purple color in the degradation system. Therefore, we attempted to mix 2,4-TDA with ABTS/ABTS^·+^, maintaining the concentrations of both substances consistent with those in the degradation system. It can be observed that mixing un oxidized ABTS with 2,4-TDA directly does not result in any color change in the solution. However, when an enzyme is introduced into the system, the mixture turns purple. This leads us to hypothesize that the enzyme oxidizes ABTS to generate ABTS^·+^, which then reacts with 2,4-TDA to produce the color change. To validate this hypothesis, we added an oxidizing agent (ammonium persulfate) to the mixture of ABTS and 2,4-TDA, and the solution indeed turned purple, confirming our speculation (Figure 3B). Additionally, we performed UV-Vis spectroscopy on the aforementioned samples. Compared to the peaks of 2,4-TDA and ABTS, no new peaks were observed in the spectra of ABTS with an oxidizing agent or ABTS with an enzyme followed by the addition of 2,4-TDA. The UV results indicated that the reaction between ABTS^·+^ and 2,4-TDA did not generate any new peaks, with the maximum absorption peak remaining at wavelength of 339 nm (Figure 3C). Furthermore, by adding varying concentrations of 2,4-TDA to the ABTS^·+^ solution, we found that the intensity of the purple color increased with higher 2,4-TDA concentrations. At the maximum absorption wavelength (OD_339_), higher 2,4-TDA concentrations resulted in greater OD_339_ values, accompanied by a deeper purple color. The OD_339_ values exhibited a strong linear correlation with 2,4-TDA concentration, with an *R*^2^ value of 0.9034 (Figure 3D).

**Figure 4:**
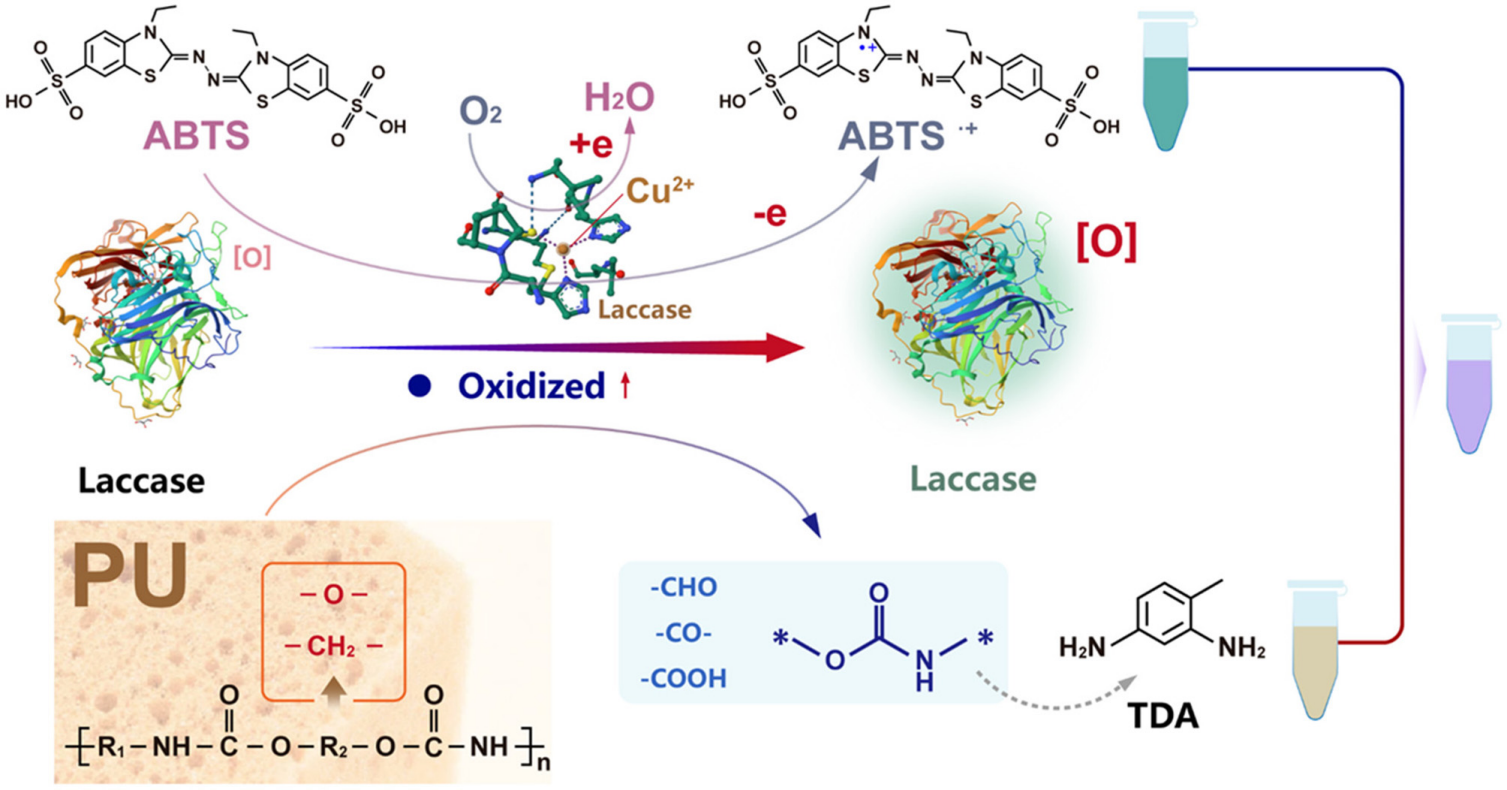
Schematic illustration of the degradation mechanism of PU by BLMS.

Therefore, we predicted the degradation process in the PU degradation by bacterial laccase-mediated system (Figure 4). First, the laccase CotA catalyzed the one-electron oxidation of the mediator molecule ABTS using its multi copper active center, generating the radical cation ABTS^·+^ while reducing O_2_ to H_2_O (Solomon et al., 1996). Subsequently, CotA could simultaneously catalyze redox reactions in both the soft segments (polyol blocks) and hard segments (isocyanate blocks) of PU, generating various oxidative degradation products including ketones, alcohols, aldehydes, carboxylic acids, esters, and ethers. Additionally, the formation of diamine monomers was detected during the degradation process. It is noteworthy that, as an oxidoreductase, laccase lacks the capability to hydrolyze urethane bonds. Therefore, we proposed that during the primary laccase-catalyzed redox reaction stage, small oligomers containing urethane linkages are initially formed. While these oligomers exhibited instability under acidic conditions, where their urethane bonds undergo spontaneous hydrolysis, ultimately decomposing into amine compounds and carbon dioxide (Chapman, 1989; Czeiszperger, 2012). Finally, the generated ABTS^·+^ reacts with the degradation product (2,4-TDA), forming a purple complex that causes the solution to turn purple.

## 4 Discussion

Bacterial laccase is a versatile oxidase with broad biological functionalities and considerable application prospects. It has been demonstrated to effectively degrade polycyclic aromatic hydrocarbons (PAHs), synthetic dyes, and lignin, while also exhibiting antimicrobial properties and being employed in pulp bleaching processes, among other industrial applications (Song et al., 2018; Zhu et al., 2020). In addition, bacterial laccase has been reported to have the ability to hydrolyze other plastics such as PE (Yao et al., 2022; Zeng et al., 2024), PS (Yuan et al., 2020). In this study, bacterial laccase CotA was reported for the first time to be capable of degrading PU. Previously reported fungal laccase isolated from *Trametes versicolor* were used to degrade PU, with weight loss reaching 12.1% and 2.3% for polyester- and polyether- PU foam, respectively (Magnin et al., 2021). The degradation capability of this fungal laccase is comparable to that of the bacterial laccase investigated in this study. Sequence alignment analysis of bacterial laccase CotA and the fungal laccase demonstrated only 31.2% amino acid sequence identity.

In this study, we observed that commercially produced polyester-CPU foams exhibited significantly lower degradability compared to laboratory-synthesized polyester-SPU. This is because the self-synthesized PU foam possessing simpler and more controllable molecular structure design with low cross-linking density, making it more vulnerable to hydrolysis. Besides, commercial PU foams incorporate highly cross-linked structures and various additives like flame retardants, antioxidants, blowing agents, etc. to enhance product durability (Skleničková et al., 2020). While these modifications improve mechanical properties and longevity, they simultaneously render the molecular chains more resistant to enzymatic recognition and cleavage. Furthermore, the dense cellular structure impedes penetration by enzymes or microorganisms, resulting in significantly reduced degradation efficiency. However, CotA demonstrated relatively poor degradation efficiency for both types of polyether-based foams. This is primarily because the ether bonds in polyether foams exhibit higher chemical stability compared to ester bonds, making them more resistant to enzymatic attack (Liu et al., 2021).

Previous studies on PU biodegradation by laccases have primarily relied on material characterization of PU samples to infer possible degradation mechanisms. In contrast, our current study has successfully identified specific degradation products (including ketones, alcohols, aldehydes, acids, esters, ethers and amines) through systematic analysis, providing direct experimental evidence that advances our understanding of the laccase-mediated PU degradation pathway beyond previous speculative approaches.

Furthermore, it is noteworthy that during the degradation process, a purple color gradually appeared in the reaction solution and on the foam surface, which was not observed during the degradation of PU mediated by other degrading enzymes. In the fungal laccase-mediated degradation of PU, HBT (1-hydroxybenzotriazole) was employed as the mediator instead of ABTS. As a result, the characteristic purple coloration of the solution was not observed. However, 2,4-TDA was detected as a degradation product. To investigate further, 2,4-TDA was reacted with ABTS^·+^, leading to the formation of a purple substance. Notably, the OD at the maximum absorption peak of this purple substance increased proportionally with higher 2,4-TDA concentrations, demonstrating a good linear correlation. Based on these observations, we propose the following reaction mechanism: laccase first oxidizes ABTS to the radical cation ABTS^**+**^, which then reacts with 2,4-TDA to generate the purple compound.

Despite these findings, UV-Vis spectral analysis did not reveal any new absorption peaks associated with the purple substance, and it appears that no new substance was formed. This absence of detectable peaks may be due to several factors, including low product concentration, incomplete reaction, or insufficient stability of the purple compound formed. To address these uncertainties, further optimization of experimental conditions and detailed analytical investigations are necessary to elucidate the structure of the purple substance and fully characterize its formation mechanism. Besides using ABTS as a mediator in the BLMS system, the appearance of the purple color can be a good indicator of the degree of degradation. Therefore, we recommend using ABTS as the mediator in the bacterial laccase-mediated degradation of PU systems, as it enables intuitive and rapid assessment of degradation efficiency through visible color changes.

## 5 Conclusion

The Bacterial Laccase-Mediated System (BLMS) constructed in this study successfully achieved the degradation of polyester-type and polyether-PU. FTIR, TGA, and GC-MS analyses confirmed the occurrence of redox reactions during the degradation process. Further studies revealed that the appearance of the purple substance was due to the degradation product 2,4-TDA react with the radical cation ABTS^·+^ which was enzymatic oxidized. In summary, the BLMS system has a degradation capability of various PU. And ABTS is recommended as a redox mediator for oxidoreductases, enabling the visualization of PU degradation through a distinct colorimetric reaction. Future studies will focus on optimizing the degradation conditions and protein engineering to further improve the degradation efficiency and practical application potential of the BLMS system.

## Data Availability

The original contributions presented in the study are included in the article/Supplementary material, further inquiries can be directed to the corresponding authors.

## Supplementary material

Supplementary Figure S1 SDS-PAGE analysis of recombinant CotA. Lane M, protein marker; Lane1, pellet after centrifugation; Lane 2, crude enzyme; Lanes 3-7, purified CotA washed with 50, 100, 200 and 300 mM imidazole, respectively.

Supplementary Table S1 Thermogravimetric analysis of polyester-CPU and polyether-CPU.
