# Supplementary figures and images for: An efficient bacterial laccase-mediated system for polyurethane foam degradation
Source: Front Microbiol. 2025 Aug 25;16:1638208. doi: 10.3389/fmicb.2025.1638208 (PMC12415013; doi:10.3389/fmicb.2025.1638208)

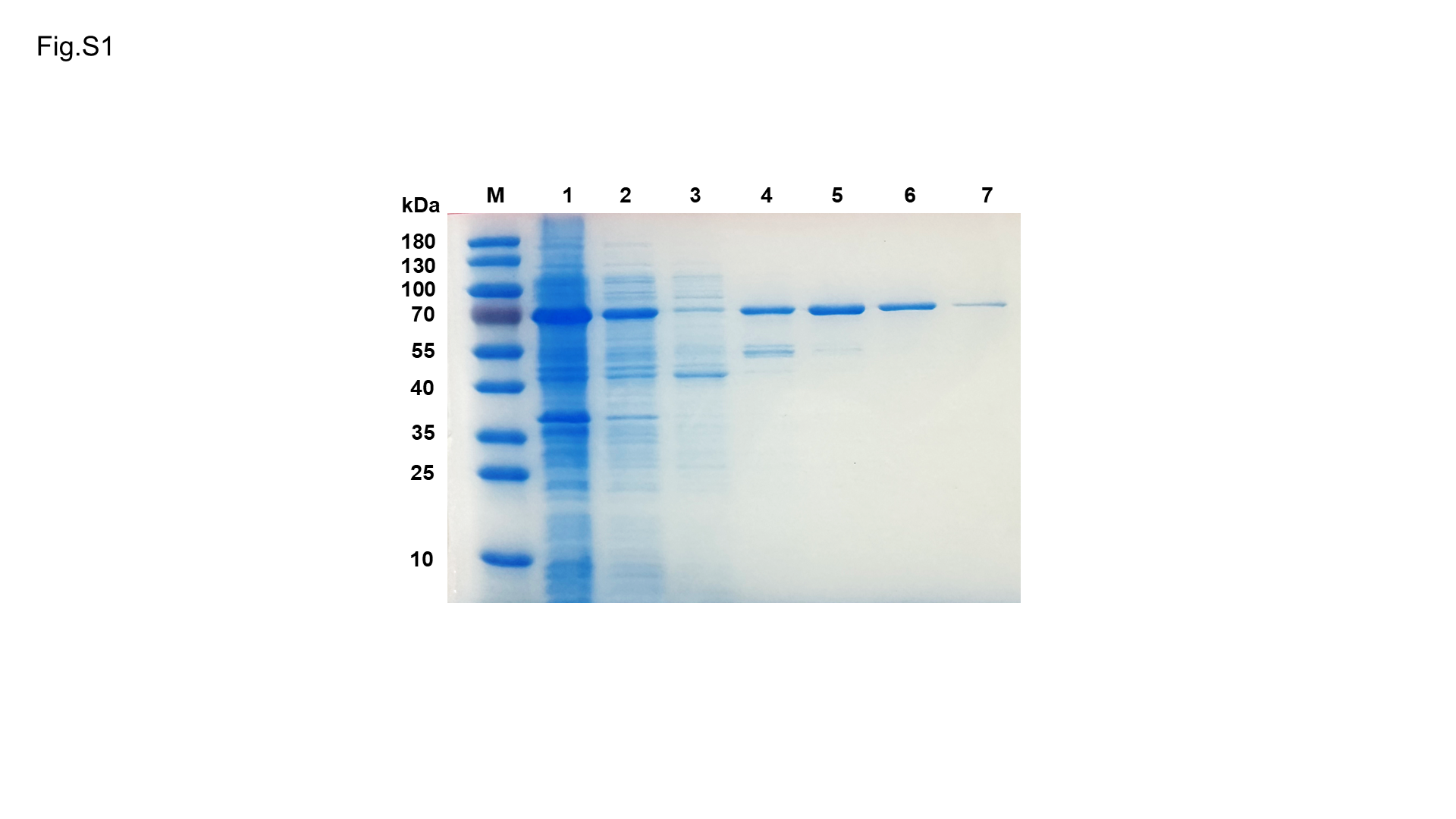

Supplement: Supplementary Figure S1 — SDS-PAGE analysis of recombinant CotA. Lane M, protein marker; Lane1, pellet after centrifugation; Lane 2, crude enzyme; Lanes 3-7, purified CotA washed with 50, 100, 200 and 300 mM imidazole, respectively. [file Supplementary_file_1.tiff]

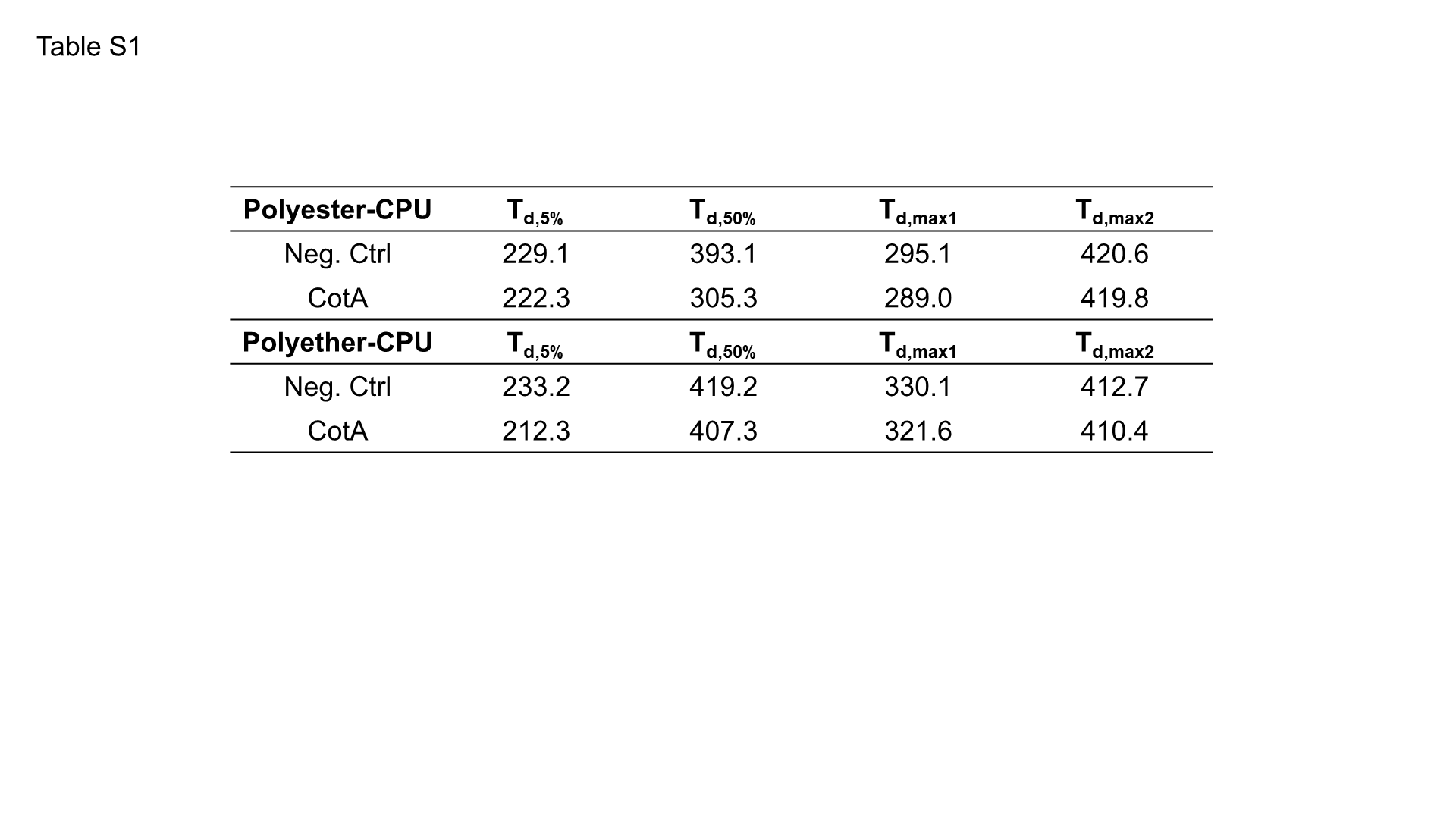

Supplement: Supplementary Table S1 — Thermogravimetric analysis of polyester-CPU and polyether-CPU. [file Supplementary_file_2.tiff]
